# Efficacy of bendamustine and rituximab in unfit patients with previously untreated chronic lymphocytic leukemia. Indirect comparison with ibrutinib in a real‐world setting. A GIMEMA‐ERIC and US study

**DOI:** 10.1002/cam4.3470

**Published:** 2020-09-24

**Authors:** Antonio Cuneo, Anthony R. Mato, Gian Matteo Rigolin, Alfonso Piciocchi, Massimo Gentile, Luca Laurenti, John N. Allan, John M. Pagel, Danielle M. Brander, Brian T. Hill, Allison Winter, Nicole Lamanna, Constantine S. Tam, Ryan Jacobs, Frederick Lansigan, Paul M. Barr, Mazyar Shadman, Alan P. Skarbnik, Jeffrey J. Pu, Alison R. Sehgal, Stephen J. Schuster, Nirav N. Shah, Chaitra S. Ujjani, Lindsey Roeker, Ester Maria Orlandi, Atto Billio, Livio Trentin, Martin Spacek, Monia Marchetti, Alessandra Tedeschi, Fiorella Ilariucci, Gianluca Gaidano, Michael Doubek, Lucia Farina, Stefano Molica, Francesco Di Raimondo, Marta Coscia, Francesca Romana Mauro, Javier de la Serna, Angeles Medina Perez, Isacco Ferrarini, Giuseppe Cimino, Maurizio Cavallari, Rosalba Cucci, Marco Vignetti, Robin Foà, Paolo Ghia, the GIMEMA, European Research Initiative (ERIC) on CLL, US study group

**Affiliations:** ^1^ Hematology Department of Medical Sciences St. Anna University Hospital Ferrara Italy; ^2^ Division of Hematological Oncology CLL Program Memorial Sloan Kettering Cancer Center New York NY USA; ^3^ Italian Group for Adult Hematologic Diseases (GIMEMA) Data Center and Health Outcomes Research Unit Rome Italy; ^4^ Department of Onco‐Hematology Hematology Unit A.O. of Cosenza Cosenza Italy; ^5^ Department of Radiological, Radiotherapeutic and Hematological Sciences Fondazione Policlinico Universitario “A. Gemelli” IRCCS Rome Italy; ^6^ Weill Cornell Medicine New York NY USA; ^7^ Center for Blood Disorders and Stem Cell Transplantation Swedish Cancer Institute Seattle WA USA; ^8^ Division of Hematologic Malignancies and Cellular Therapy Duke University Durham NC USA; ^9^ Taussig Cancer Institute Cleveland Clinic Cleveland OH USA; ^10^ Columbia University Medical Center New York NY USA; ^11^ Peter McCallum Cancer Centre University of Melbourne Melbourne Victoria Australia; ^12^ Department of Hematologic Oncology and Blood Disorders Levine Cancer Institute Carolinas Healthcare System Charlotte NC USA; ^13^ Dartmouth‐Hitchcock Medical Center Lebanon NH USA; ^14^ Wilmot Cancer Institute University of Rochester Medical Center Rochester NY USA; ^15^ Fred Hutchinson Cancer Research Center Seattle Cancer Care Alliance Seattle WA USA; ^16^ Lymphoproliferative Disorders Program Novant Health Cancer Institute Charlotte NC USA; ^17^ SUNY Upstate Medical University SUNY Upstate Medical University Syracuse NY USA; ^18^ University of Pittsburgh Pittsburgh PA USA; ^19^ Division of Hematology and Oncology University of Pennsylvania Philadelphia PA USA; ^20^ Division of Hematology & Oncology Medical College of Wisconsin Milwaukee WI USA; ^21^ Hematology Unit IRCCS Policlinico San Matteo Pavia Italy; ^22^ Hematology and Transplant Unit San Maurizio Hospital Azienda Sanitaria dell'Alto Adige Bolzano Italy; ^23^ Hematology and Clinical Immunology Department of Medicine University of Padua Padua Italy; ^24^ Department of Medicine Department of Hematology First Faculty of Medicine Charles University and General University Hospital Prague Czech Republic; ^25^ Oncology Unit Cardinal Massaia Hospital Asti Italy; ^26^ Hematology Niguarda Cancer Center ASST Grande Ospedale Metropolitano Niguarda Milan Italy; ^27^ Hematology Azienda USL‐IRCCS Reggio Emilia Italy; ^28^ Division of Hematology Department of Translational Medicine University of eastern Piedmont Novara Italy; ^29^ Department of Internal Medicine ‐ Hematology and Oncology University Hospital Brno and Faculty of Medicine Masaryk University Brno Czech Republic; ^30^ Hematology Department Fondazione IRCCS Istituto Nazionale Tumori Milano Italy; ^31^ Hematology Unit A. Pugliese Hospital Azienda Ospedaliera Pugliese Ciaccio Catanzaro Italy; ^32^ Catania Università di Catania Cattedra di Ematologia Catania Italy; ^33^ Division of Hematology A.O.U. Città della Salute e della Scienza di Torino and Department of Molecular Biotechnology and Health Sciences University of Torino Torino Italy; ^34^ Hematology Department of Translational and Precision Medicine "Sapienza" University Rome Italy; ^35^ Hematology Unit Hospital Universitario Madrid Spain; ^36^ Hospital Costa del Sol Marbella Spain; ^37^ Hematology Department of Cell Therapy and Hematology University Hospital Verona Italy; ^38^ Department of Translational and Precision Medicine University “La Sapienza” UOC di Ematologia con Trapianto Ospedale S. Maria Goretti Latina Italy; ^39^ Strategic Research Program on CLL Division of Experimental Oncology IRCCS Ospedale San Raffaele Università Vita‐Salute San Raffaele Milan Italy

**Keywords:** bendamustine, chronic lymphocytic leukemia, ibrutinib, real‐world analysis, unfit patients

## Abstract

Limited information is available on the efficacy of front‐line bendamustine and rituximab (BR) in chronic lymphocytic leukemia (CLL) with reduced renal function or coexisting conditions. We therefore analyzed a cohort of real‐world patients and performed a matched adjusted indirect comparison with a cohort of patients treated with ibrutinib. One hundred and fifty‐seven patients with creatinine clearance (CrCl) <70 mL/min and/or CIRS score >6 were treated with BR. The median age was 72 years; 69% of patients had ≥2 comorbidities and the median CrCl was 59.8 mL/min. 17.6% of patients carried TP53 disruption. The median progression‐free survival (PFS) was 45 months; TP53 disruption was associated with a shorter PFS (*P* = 0.05). The overall survival (OS) at 12, 24, and 36 months was 96.2%, 90.1%, and 79.5%, respectively. TP53 disruption was associated with an increased risk of death (*P* = 0.01). Data on 162 patients ≥65 years treated with ibrutinib were analyzed and compared with 165 patients ≥65 years treated with BR. Factors predicting for a longer PFS at multivariable analysis in the total patient population treated with BR and ibrutinib were age (HR 1.06, 95% CI 1.02‐1.10, *P* < 0.01) and treatment with ibrutinib (HR 0.55, 95% CI 0.33‐0.93, *P* = 0.03). In a post hoc analysis of patients in advanced stage, a significant PFS advantage was observed in patient who had received ibrutinib (*P* = 0.03), who showed a trend for OS advantage (*P* = 0.08). We arrived at the following conclusions: (a) BR is a relatively effective first‐line regimen in a real‐world population of unfit patients without TP53 disruption, (b) ibrutinib provided longer disease control than BR in patients with advanced disease stage.

## Graphical abstract

Bendamustine and Rituximab was a relatively effective first‐line regimen in real‐world untreated CLL patients with reduced renal function or coexisting conditions without TP53 disruption.In a matched‐adjusted indirect comparison with a cohort of CLL patients treated upfront, ibrutinib provided longer PFS than bendamustine and rituximab in those with advanced stage.

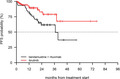

## 1 INTRODUCTION

With 20,720 estimated new cases and 3930 deaths in 2019 in the United States, chronic lymphocytic leukemia (CLL) is the most frequent leukemia in western countries.^1^ Because the median age at diagnosis is around 70 years and many patients carry one or more comorbidities, the most effective chemoimmunotherapy (CIT) regimen, fludarabine, cyclophosphamide, and rituximab (FCR), cannot be safely administered, thus making other options like a combination of the anti CD20 monoclonal antibody rituximab with bendamustine (BR) or chlorambucil^2^ a widely employed therapeutic strategy in the clinical practice. The efficacy and safety of these combinations in the front‐line setting have been documented in phase‐2 and phase‐3 trials including fit patients randomized to either FCR or BR,^3^, ^4^, ^5^ or patients deemed ineligible for fludarabine due to coexisting conditions and/or reduced renal function who were treated with chlorambucil and anti CD20 monoclonal antibodies.^6^, ^7^, ^8^, ^9^, ^10^ The BR combination was also tested in a limited number of patients not deemed fit to receive fludarabine‐based regimens at physician's discretion^9^ and in older patients with preserved or moderately impaired renal function.^11^

In recent years, evidence has been provided that a longer progression‐free survival (PFS) can be achieved with ibrutinib than with CIT in previously untreated CLL^10^, ^11^, ^12^ and expert opinions recommended ibrutinib upfront, especially in older patients with CLL and in younger patients with *TP53* disruption or less favorable unfavorable immunogenetic characteristics, that is, unmutated configuration of the variable portion of the immunoglobulin (*IGHV*) gene.^13^, ^14^, ^15^

Interestingly, an observational study of CIT in the "real‐world" setting showed that these regimens may prove safe and effective,^16^, ^17^, ^18^ even though dose reductions may occur commonly in clinical practice and may impact on outcome.^19^ Likewise, the rates of ibrutinib discontinuation and survival are likely to be worse in a real‐world setting than in a clinical trial possibly due to inclusion of patients with poorer performance status and more comorbidities.^20^, ^21^, ^22^ A matched‐adjusted indirect comparison of patients who had received BR or ibrutinib as first salvage treatment outside of clinical trials showed no OS difference.^17^

Because limited information is available on the efficacy and safety of BR in unfit patients, we analyzed the efficacy of this regimen in a cohort treated outside clinical trials and we carried out an indirect comparison with a cohort of older patients treated with ibrutinib front‐line in a real‐world setting.^23^

## 2 METHODS

### 2.1 Patients

The patients included in this report were retrospectively selected as outlined below from a cohort of patients treated front‐line with BR between 2008 and 2014 at GIMEMA and European Research Initiative on CLL (ERIC) centers and from a cohort of CLL patients treated front‐line with ibrutinib at 20 community and academic US centers.^16^, ^22^

### 2.2 BR regimen in unfit CLL

Inclusion criteria in this analysis were (a) CLL progression according to the NCI criteria^24^ (b) no previous treatment for CLL, (c) creatinine clearance <70 mL/min and/or CIRS > 6, that is unfit patients as previously defined,^6^ (d) and received at least 1 day of treatment with the BR regimen.^25^ Patients were excluded from the study if they had transformation of CLL into Richter's syndrome before starting treatment, known HIV infection, active and uncontrolled HCV and/or HBV infections. Treatment response and disease progression were assessed according to the NCI criteria.^24^ The primary endpoint was 12‐month progression‐free survival (PFS). Secondary endpoints were overall response rate (ORR), time to next anti‐leukemic treatment (TTNT), and overall survival (OS) as previously defined.^17^ Safety data were reported according to the NCI Common Terminology Criteria for Adverse Events version 4.0. The study was registered at ClinicalTrials.gov (NCT02491398). The study was approved by the institutional review board of each participating institution.

### 2.3 Indirect comparison with ibrutinib

For the purpose of this analysis, we retrieved pertinent data by reviewing clinical charts, electronic medical records, and related databases in patients ≥65 years enrolled in the GIMEMA ERIC study who had received BR in first line and in patients ≥65 years of a US study who had received single agent ibrutinib.^22^ The patients with del(17p) or *TP53* mutations were excluded. The endpoints for this analysis were the PFS and OS.

### 2.4 Statistical analysis

The analyses were performed following the intention‐to‐treat principle as previously described.^17^ All analyses were performed using the SAS software (version 9.4 or later); all tests were two‐sided, at a significance level of 0.05. Study data were collected and managed using REDCap electronic data capture tools.^26^ The data that support the findings of this study are available from the corresponding author upon reasonable request.

## 3 RESULTS

### 3.1 Treatment with BR in unfit patients

One hundred fifty‐seven patients treated at 31 centers (24 GIMEMA centers and 7 ERIC centers) were included. The demographic data at baseline have been outlined in Table 1. The median age was 72 years; 80.9% of patients was >65 years; 69.2% had 2 or more comorbidities, the median creatinine clearance was 59,8 mL/min and 58.9% had Binet stage B‐C. Fifty‐one percent (data available in 56.7% of the patients) had an unmutated *IGHV* gene configuration, 17.6% carried 17p‐ and/or *TP53* mutation (data available in 83.4% of the patients).

**TABLE 1:** Baseline characteristics of 157 patients in the bendamustine and rituximab (BR) cohort

Variable (n. of patients)	N. of patients (%)
age ≤ 65/>65 years (n = 157)	30 (19.1)/127 (80.9)
median age (range) (n = 157)	72.5 (39‐89)
stage^a^ early/intermediate‐advanced (n = 129)	53 (41.1)/76 (58.9)
gender male/female (n = 157)	95 (60.5)/62 (39.5)
ECOG PS 0/1/ ≥2 (n = 154)	68 (43.3)/70 (44.6)/ 16 (10.2)
N. of comorbidities 0‐1/≥ 2 (n = 156)	48 (30.8)/108 (69.2)
Median Cr Cl ml/min (range) (n = 157)	59.8 (22.0−137.0)
beta 2 microglobulin ≥ 3.5/<3.5 mg/L (n = 113)	94 (83.2)/19 (16.8)
*IGHV* Mutated/Unmutated (n = 89)	44 (49.4)/45 (50.6)
17p‐ and/or *TP53* mutated yes/no (n = 131)	23 (17.6)/108 (82.4)
FISH abns 13q‐/+12/11q‐/17p‐/normal (n = 111)	34 (30.6)/24 (21.6)/10 (9.0)/7 (6.3)/36 (32.4)

Note

Legend: ECOG PS, Eastern Cooperative Oncology Group Performance Status; Cr Cl, Creatinine clearance.

^a^ Early: Binet A.

One hundred and fourteen of 157 patients (73.1%) received the planned number of cycles; treatment was discontinued as a result of toxicity in 18% patients (n = 28), withdrawal of consent in 2% (n = 3), or other reasons in 7% (n = 11). The number of cycles actually administered to patients who discontinued treatment was ≥4 in 71% of cases. The starting dose of bendamustine was 90 mg/m^2^ in 45.8% of the patients, 70 mg/m^2^ in 33.1% of the patients and 50‐70 mg/m^2^ in 21.1% of the patients. A dose reduction >10% of the planned dose of bendamustine was recorded in 24.2% of cases; treatment delay occurred in 22.9% of patients.

Second‐line treatment was offered at symptomatic progression according to local guidelines.

### 3.2 Efficacy

The ORR to BR was 91.7%. The probability of attaining a response was significantly lower in patients with del(17p)/*TP53* mutations as compared to patients without del(17p)/*TP53* mutations (50% vs 85.1%, *P* = 0.02). Other variables had no significant impact on the ORR (Table S2).

**FIGURE 1:**
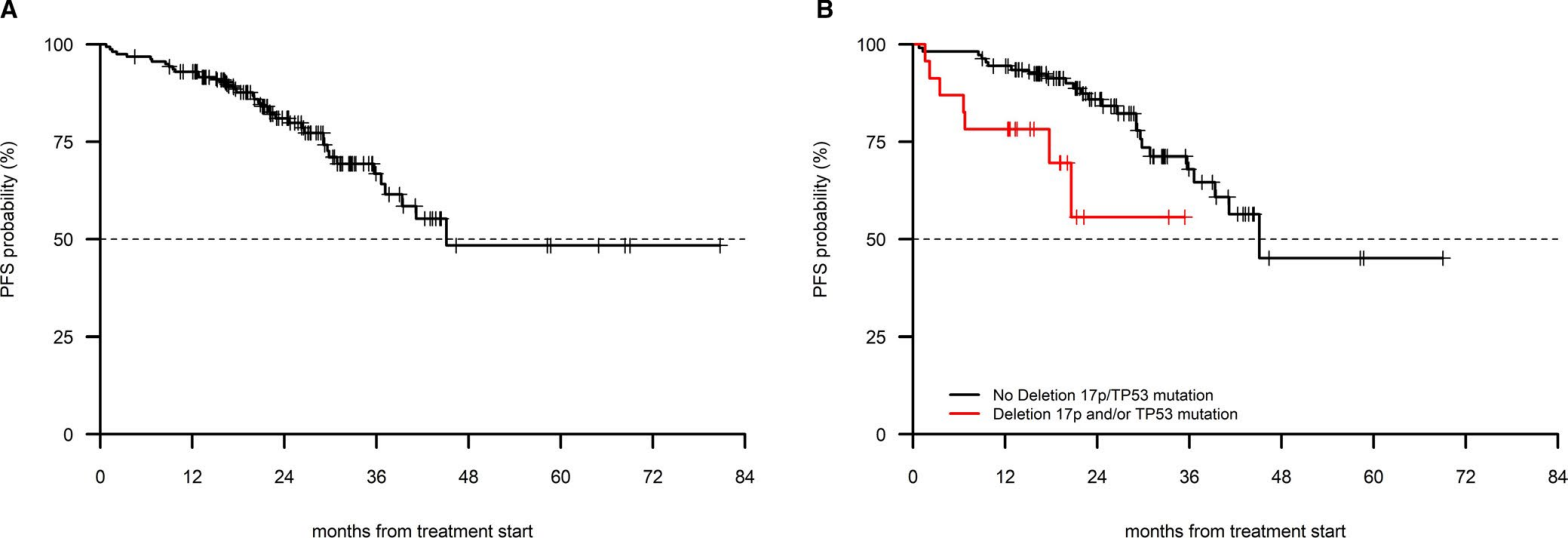
PFS in 157 patients treated with BR (A) in the entire cohort, (B) in patients with and without 17p‐/TP53 mutation

The median PFS was 45 months (Figure 1(A)), with a 12‐month PFS rate of 93% (95% CI 89.0%‐97.1%). The estimated PFS at 24 and at 36 months were 81% (95% CI 74.5%‐88.2%) and 66.8% (95% CI 74.5%‐88.2%), respectively. Factors associated with a shorter PFS in univariate analysis were represented by del(17p)/*TP53* mutations, ECOG performance status and by the presence of 2 or more comorbidities, as shown in Table 2, whereas an intermediate/advanced stage and an IGHV*‐*unmutated status were of borderline significance. Age (cut‐off 65 years) and other clinicobiologic parameters had no impact on PFS. del(17p)/*TP53* mutations represented the only adverse parameter in multivariable analysis (Table 2, Figure 1(B)).

**TABLE 2:** Progression‐free survival (PFS): univariate and multivariate analyses in the bendamustine and rituximab (BR) cohort

	Univariate		Multivariate	
	HR (95% CI)	*P*	HR (95% CI)	*P*
Age > 65 vs ≤ 65 years	1.85 (0.66‐5.24)	0.24		
Binet B‐C vs Binet A	2.16 (0.95‐4.92)	0.07		
beta 2 microglobulin ≥ 3.5 vs < 3.5 mg/L	1.64 (0.57‐4.70)	0.36		
*IGHV* unmutated vs mutated	2.29 (0.92‐5.69)	0.07		
17p‐ and/or *TP53* mutated yes vs no	3.14 (1.29‐7.63)	0.01	2.52 (1.00‐6.39)	0.05
Gender female vs male	0.60 (0.31‐1.17)	0.13		
ECOG 0 vs 1	1.64 (0.78‐3.46)	0.19		
ECOG 0 vs ≥ 2	3.12 (1.25‐7.81)	0.02		
Comorbidities 0‐1 vs ≥ 2	2.91 (1.14‐7.45)	0.03		
Creatinine clearance ≤ 70 vs > 70 mL/min	1.76 (0.69‐4.54)	0.24		

A second‐line treatment was administered to 3.2% (95% CI 1.2%‐6.9%), 8.3% (95% CI 4.3%‐13.9%), and 21.5% (95% CI 13.1%‐31.1%) of patients at 12, 24, and 36 months, respectively (Figure S1). A shorter TTNT was associated with del(17p)/*TP53* mutation (*P* < 0.01; Table S2).

**FIGURE 2:**
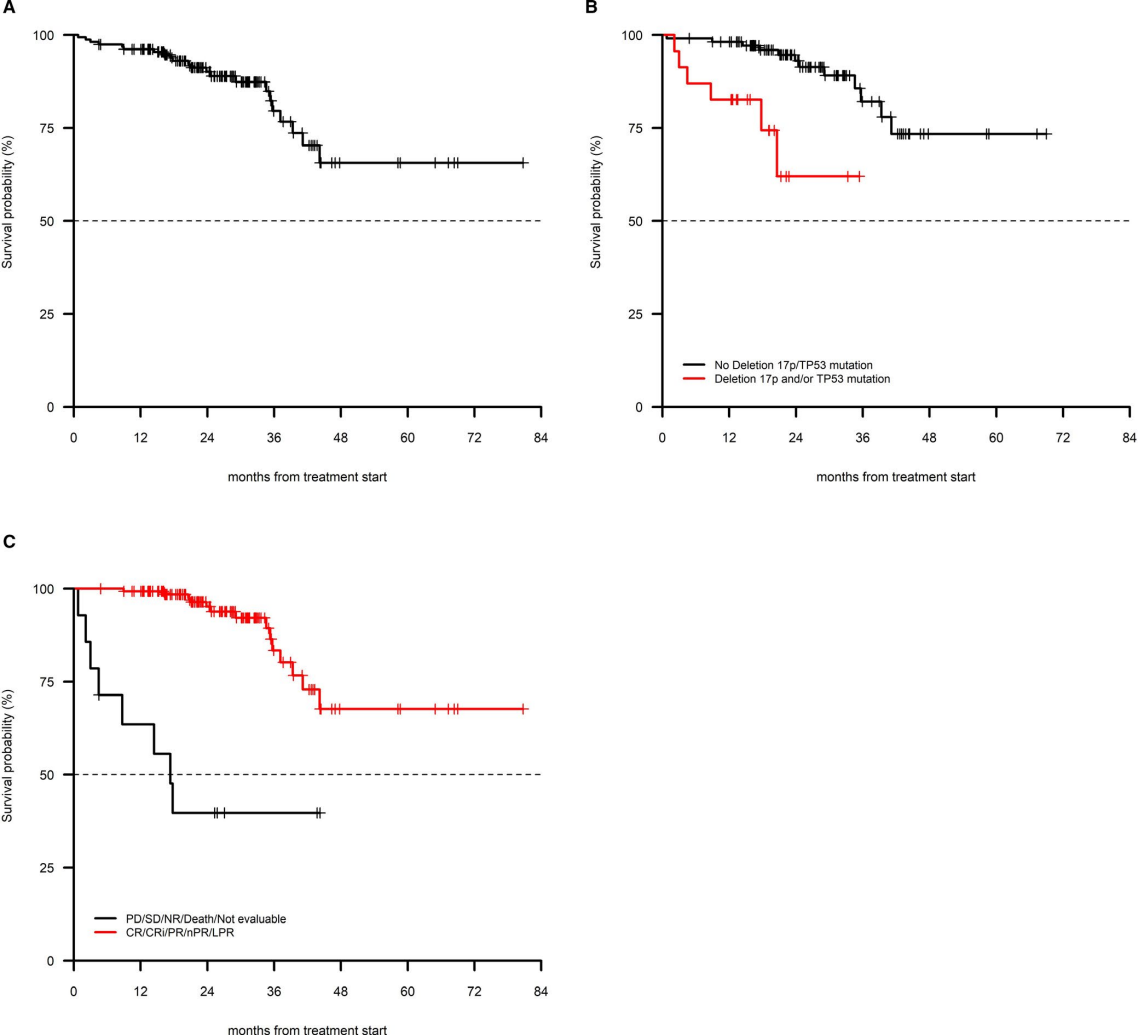
OS in 157 patients treated with BR (A) in the entire cohort, (B) in patients with and without 17p‐/TP53 mutation, (C) in patients who had a response to treatment vs patients who did not respond to treatment

The OS at 12, 24, and 36 months were 96.2% (95% CI 93.2%‐99.2%), 90.1% (95% CI 85.0%‐95.5%), and 79.5% (95% CI 70.0%‐90.5%), respectively, with a median OS that was not reached with a 26‐month median follow‐up (Figure 2). The presence of del(17p)/*TP53* mutation (*P* < 0.01) and lack of response to treatment (*P* < 0.01) were associated with a shorter OS in univariate as well as in multivariate analysis (Table 3). Other clinicobiologic parameters had no impact on OS. Twenty‐two patients died (n = 2 due to CLL, 9.1%; n = 12 infection with or without active CLL, 54.6%; n = 3 second primary tumors, 13.6%; 1 each cardiac disease and sudden death). In three patients, the cause of death was not reported.

**TABLE 3:** Overall survival (OS): univariate and multivariate analyses in the bendamustine and rituximab (BR) cohort

	Univariate		Multivariate	
	HR (95% CI)	*P*	HR (95% CI)	*P*
Age > 65 vs ≤ 65 years	3.95 (0.53‐29.50)	0.18		
Binet B‐C vs Binet A	2.42 (0.79‐7.41)	0.12		
β2 microglobulin ≥ 3·0.5 vs < 3.5 mg/L	4.11 (0.55‐30.92)	0.17		
IGHV unmutated vs mutated	0.81 (0.22‐3.05)	0.76		
17p‐ and/or *TP53* mutated yes vs no	6.14 (2.08‐18.13)	<0.01	4.47 (1.37‐14.56)	0.01
NR vs CR or PR	8.43 (3.52‐20.19)	<0.01	15.21 (5.72‐40.74)	<0.01
Gender female vs male	0.68 (0.29‐1.64)	0.39		
ECOG 0 vs 1	2.28 (0.79‐6.59)	0.13		
ECOG 0 vs ≥ 2	4.90 (1.48‐16.19)	0.01		
Comorbidities 0‐1 vs ≥ 2	1.79 (0.60‐5.29)	0.29		
Creatinine clearance ≤ 70 vs > 70 mL/min	3.02 (1.01‐9.09)	0.05		
no‐aberrations vs 13q	0.74 (0.12‐4.45)	0.74		
11q‐ vs 13q	3.18 (0.52‐19.37)	0.21		
+12 vs 13q	3.88 (0.90‐16.71)	0.07		
17p‐ vs 13q	11.29 (2.08‐61.14)	<0.01		

Legend: NR, no response; CR, complete response; PR, partial response.

### 3.3 Safety

Forty‐two percent of the BR patients reported at least one grade 3‐4 adverse event. Overall, cytopenias were recorded in 34% of patients. Grade 3‐4 neutropenia occurred in 24% of cases. Grade 3‐4 infections including febrile neutropenia were recorded in 11% of patients. One case of fatal infection was reported. Grade 3‐4 rash and/or dermatitis were reported 3% of patients.

### 3.4 Comparison of BR in the GIMEMA‐ERIC dataset and ibrutinib in the US dataset

Data on 165 and 162 older patients without del(17p)/*TP53* aberrations treated with BR and ibrutinib, respectively, were analyzed. The median follow‐up in the BR and in the ibrutinib cohorts was 29 months (95% CI 26‐31) and 13 months (95% CI 10‐14), respectively. As shown in Table 4, the two cohorts were comparable in terms of age and frequency of del(11q). Patients with advanced Rai stage were more frequently represented in the ibrutinib cohort than in the BR cohort, and the interval between diagnosis and treatment was significantly longer in the ibrutinib cohort.

**TABLE 4:** Baseline characteristics of the bendamustine and rituximab (BR) and the ibrutinib cohorts

	BR n = 165 (%)	ibrutinib = 162 (%)	*P*
Age ≤ 70/ >70 years	53 (32.1)/ 112 (67.9)	53 (32.7)/ 109 (67.3)	1.00
gender Male/ Female	106 (64.2)/ 59 (35.8)	102 (63.0)/ 60 (37.0)	0.90
Time dx‐trx^a^ < 36/≥36 months	99 (60.0)/ 66 (40.0)	69 (42.6)/ 93 (57.4)	<0.01
RAI^b^ stage 0‐2/ 3‐4	79 (63.2)/ 46 (36.8)	59 (38.1)/ 96 (61.9)	<0.01
del11q no/ yes	101 (89.4)/ 12 (10.6)	125 (87.4)/ 18 (12.6)	0.77

^a^ Interval between diagnosis and treatment.

^b^ The Rai staging system was used here because it was adopted in the US cohort.

Factors associated with a longer PFS in multivariate analysis in the combined patient population treated with BR and ibrutinib were represented by age as a continuous variable and by treatment with ibrutinib (Table 5). Age was the only factor predicting for shorter OS at multivariable analysis (HR 1.10, 95% CI 1.04 ‐ 1.15, *P* < 0.01).

**TABLE 5:** Univariate and multivariable analysis of PFS in the total patient population treated with bendamustine and rituximab (BR) and ibrutinib

Variable	univariate	*P*	Multivariate	*P*
	HR (95% CI)		HR (95% CI)	
Age (as continuous variable)	1.05 (1.02‐1.09)	<0.01	1.06 (1.02‐1.10)	<0.01
RAI 3‐4 vs 0‐2	1.09 (0.66‐1.79)	0.73	—	—
11q‐ yes vs no	2.13 (1.04‐4.37)	0.04	—	—
Time dx‐trx^a^ (continuous variable)	1.00 (0.99‐1.00)	0.62	—	—
interval dx‐trx^a^ ≥ 36 vs < 36 months	0.89 (0.57‐1.38)	0.61	—	—
Ibr vs. BR	0.60 (0.36‐1.01)	0.05	0.55 (0.33‐0.93)	0.03

^a^ Interval between diagnosis and treatment

**FIGURE 3:**
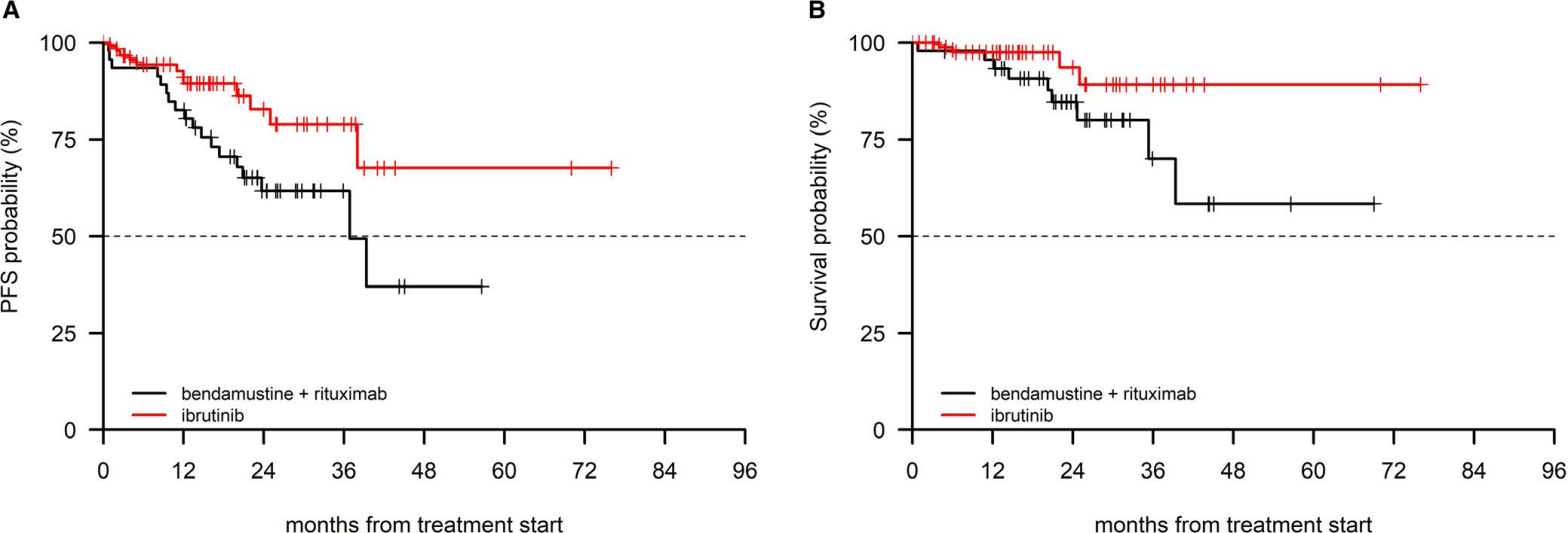
PFS (A) and OS (B) in the BR and in the ibrutinib cohort in (Rai stage III‐IV)

**FIGURE 4:**
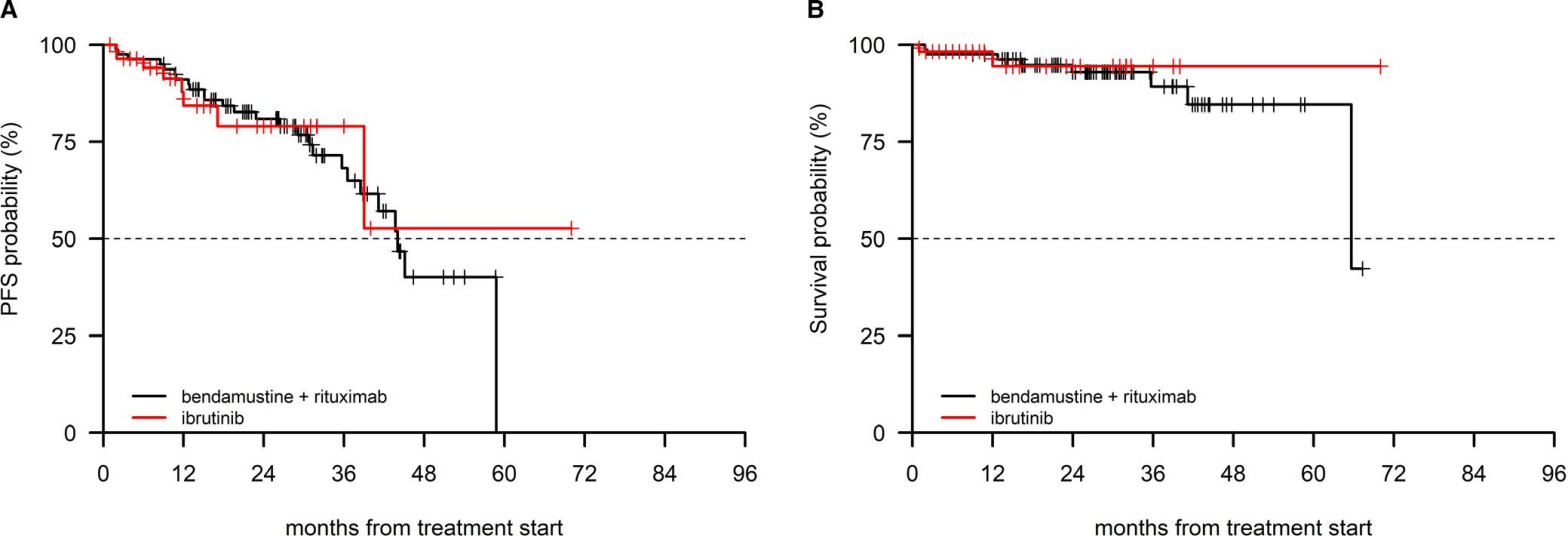
PFS (A) and OS (B) in the BR and in the ibrutinib cohort in (Rai stage 0‐II)

In a post hoc analysis including patients in advanced stage (ie Rai III‐IV), the ibrutinib cohort (n = 96 patients), and the BR cohort (n = 46 patients) were comparable in terms of age (cut‐off 70 years) and interval between diagnosis and first‐line treatment (cut‐off 36 months), as shown in Table 6. A significant PFS advantage was observed in the ibrutinib cohort (*P* = 0.03), which also showed a trend for an advantage in terms of OS (*P* = 0.08, Figure 3(A) and (B)). Patients in early/intermediate stage in the BR (n = 79) and in the ibrutinib cohort (n = 59) had similar age and similar interval between diagnosis and first‐line treatment characteristics (Table 6). No difference was noted in terms of PFS (*P* = 0.89) and OS (*P* = 0.66) in these patients (Figure 4(A) and (B)).

**TABLE 6:** Baseline characteristics in patients with advanced (Rai III‐IV) and early/intermediate stage (Rai 0‐II) in the bendamustine and rituximab (BR) and ibrutinib cohorts

	Advanced stage				Early/intermediate stage		*P*
		BR n = 46 (%)	ibrutinib n = 96 (%)	*P*	BR n = 79 (%)	ibrutinib n = 59 (%)	
Age	≤70 years	16 (34.8)	27 (28.1)	0.54	31 (39.2)	22 (37.3)	0.96
	>70 years	30 (65.2)	69 (71.9)		48 (60.8)	37 (67.2)	
Gender	Male	31 (67.4)	58 (60.4)	0.54	49 (62.0)	38 (64.4)	0.91
	Female	15 (32.6)	38 (39.6)		30 (38.0)	21 (35.6)	
Time dx‐trx^a^	<36 months	25 (54.3)	35 (36.5)	0.07	50 (63.3)	30 (50.8)	0.20
	≥36 months	21 (45.7)	61 (63.5)		29 (36.7)	29 (49.2)	
del11q	No	26 (78.8)	77 (88.5)	0.29	55 (96.5)	41 (83.7)	0.06
	Yes	7 (21.2)	10 (11.5)		2 (3.5)	8 (16.3)	

^a^ Time dx‐trx: Interval between diagnosis and treatment.

## 4 DISCUSSION

This is the first robust report of the efficacy and safety of BR in unfit patients treated outside clinical trials, as these patients were usually enrolled in trials using chlorambucil and an anti CD20 monoclonal antibody.^6^, ^27^ Even though chlorambucil is the preferred option as chemotherapy backbone in this subset of CLL,^15^ BR is a widely adopted front‐line regimen in the clinical practice for elderly CLL patients with coexisting conditions.^28^, ^29^

Even though this study is not based on a registry reporting the efficacy of BR and ibrutinib in all the incident CLL cases and accepting the limitations of a retrospective analysis, to ensure accuracy in data collection we encouraged each participating center to include all patients who started BR and we performed consistency checks on each case report form. Furthermore, we included, in addition to PFS, objective measures of efficacy such as OS and TTNT. Because bone biopsy was not mandatory, we were not able to assess complete response.

Our BR cohort resembled closely patients with CLL treated in the daily clinical practice in terms of age, creatinine clearance, performance status and comorbidities.^30^ The percentage of patients with early stage disease in our analysis (41.1%) was higher than in GCLLSG and in UK trials, where 23% and 33% of the patients, were in Binet stage A, respectively.^6^, ^31^ This finding reflects the tendency to initiate treatment in the presence of a short lymphocyte doubling time or of symptomatic disease in this unfit patient population.

The proportion of patients who completed the planned therapy in our analysis (73.1%) is in line with the data form the MABLE trial that compared chlorambucil and rituximab with the BR regimen in a fludarabine‐ineligible CLL population.^9^

Grade 3‐4 infections in this study (11%) occurred at a similar frequency as in clinical trials (7.7%‐19%).^9^, ^11^, ^25^ We observed a lower incidence of grade 3‐4 cytopenia (34%) compared to other studies reporting a 52.1%‐56% incidence of cytopenia due to fact that many investigators did not perform a blood count at the nadir time point.

With a median PFS of 45.1 months and a projected OS rate at 24 and 36 months of 90.1% and 79.5%, respectively, our data show that BR is an effective front‐line treatment option for an unfit patient population treated outside of clinical trials. The only baseline characteristics with a significant negative impact on the efficacy endpoints in this study were represented by the *TP53* disruption and, interestingly, failure to respond to BR was an independent dynamic prognostic factor for OS, a finding probably reflecting the lack of effective salvage treatment for a proportion of patients during the study period. The *IGHV*‐unmutated configuration was associated with a shorter PFS, that was of borderline significant probably as a consequence of the limited number of patients assessed.

In our analysis PFS is similar to that observed in the MABLE trial (median 39.6 months).^9^ Interestingly, a median PFS of 33.9 months was observed in the subset of patients with creatinine clearance <70 in a phase‐2 study^25^ and a median PFS of 43 months with a 74% PFS rate at 24 months was reported in a trial enrolling older CLL patients.^11^ Notably, OS was similar in our analysis and in prospective trials which reported an OS rate of approximately 80% at 36 months and an OS rate of 90%‐95% at 24 months.^11^, ^25^

It is worth noting that chlorambucil and rituximab produced an 87% ORR with an OS rate at 48 and 60 months of 86.1% (95% CI: 79.4‐93.5) and 81.2% (95% CI: 72.4‐91.2), respectively, in elderly patients treated outside clinical trials.^18^ Likewise, an 80.3% ORR and estimated 2‐year OS of 88% was recorded by the Israeli end ERIC group in elderly patients who received chlorambucil and obinutuzumab, without unexpected adverse events.^32^ Taken together, these data show that the efficacy and safety of different CIT regimens in routine clinical practice are consistent with those reported in clinical trials.

Because there is evidence that treatment continuation and efficacy with ibrutinib may be inferior outside of clinical trials,^21^, ^22^ and given the growing importance of real world evidence which can provide invaluable information to supplement randomized clinical studies,^33^ we elected to compare PFS and OS in a cohort treated with ibrutinib in a real‐world experience in the USA^23^ with a similar patient population treated with BR at the GIMEMA‐ERIC centers.

Although the two groups were treated in different time periods with consequent difference in follow‐up time, we performed an indirect comparison trying to minimize the effect of the heterogeneity of patient populations.

First, we documented that in the total study population (excluding patients with 17p‐) age and treatment with ibrutinib were associated with a longer PFS in multivariate analysis.

When restricting our analysis to patients with advanced stage (Rai III‐IV) with comparable baseline characteristics, we were able to show that ibrutinib was associated with a longer PFS and a trend of longer OS as compared with BR, whereas no difference was observed in patients with early intermediate stage. Though caution should be exercised when interpreting these data, as they were obtained in quite a small number of patients treated outside of clinical trial, it is noteworthy that similar findings were reported in a randomized prospective study.^34^

Taken together our data show that (a) BR is a safe and effective first‐line regimen in a real‐world cohort of unfit CLL patients, with the exception of patients with unfavorable genetic characteristics (ie del(17p)/*TP53* aberrations and *IGHV*‐unmutated configuration, (b) there are no appreciable differences in terms of efficacy and safety between BR in clinical trials that enrolled older patients with CLL and this real world experience on unfit patients with reduced renal function and with coexisting conditions and, (ci) and ibrutinib provided more durable disease control than BR in the front‐line setting in patients treated outside of clinical trials, especially in patients with advanced disease stage.

While providing evidence that BR is an effective first‐line regimen in unfit patients, these data reinforce the notion that ibrutinib has an established place in the front‐line treatment of older patients with CLL.^11^, ^15^, ^35^ These findings may have practical implications in the definition of treatment algorithms, especially in those countries with restrictions to the use of ibrutinib or other oral agents.^36^, ^37^

## CONFLICT OF INTEREST

- Antonio Cuneo: advisory board and lecturing for Janssen, Gilead, Abbvie, Roche.
- Anthony R. Mato: consultancy for TG Therapeutics (in addition DSMB), Abbvie, Pharamacyclics, Johnson & Johnson, Regeneron, Astra Zeneca, and Celgene and research funding from TG Therapeutics, Abbvie, Pharamacyclics, Johnson & Johnson, Regeneron, Portola, DTRM, and Acerta.
- Gian Matteo Rigolin: lecturing for Abbvie, Gilead and research funding from Gilead.
- Luca Laurenti advisory board and lecturing for Janssen, Gilead, Abbvie, Roche and Astra Zeneca
- John N. Allan Advisory board/Consultant for Sunesis, PCYC, Abbvie, Genentech and research funding from Janssen, Genentech
- John M. Pagel: Consultancy for Astrazeneca, Pharmacyclics, and Gilead.
- Constantine S. Tam honorarium and research funding from Janssen.
- Paul M. Barr: consultancy for from Abbvie/Pharmacyclics, Gilead, Janssen, TG therapeutics, AstraZeneca, Celgene, Morphosys, Seattle Genetics.
- Alan P. Skarbnik: consultancy for Abbvie, Pharmacyclics, Celgene, Kite, AstraZeneca, Genentech, Seattle Genetics; Speakers Bureau for Abbvie, Pharmacyclics, Celgene, Kite, Gilead Sciences, Jazz Pharma, Beigene, AstraZeneca, Genentech, Seattle Genetics, Verastem, Novartis; Stock Ownership in COTA Healthcare.
- Nirav N. Sha: honoraria, travel support, and research funding from Miltenyi Biotec, honoraria and travel support from Incyte, and Celgene; advisory boards for Kite, Celgene, and Cellectar; research support for clinical trials from BMS
- Chaitra S. Ujjani: research support from Pharmacyclics, Astra Zeneca and Abbvie; consulting for Astra Zeneca and Abbvie.
- Lindsey Roeker: ASH grant funding for work outside of this manuscript, minority ownership interest in AbbVie and Abbott Laboratories
- Gianluca Gaidano: Advisory Boards for Janssen, Abbvie, Astra‐Zeneca, Sunesis
- Michael Doubek: Honoraria and Research grants from Roche, AbbVie, AOP Orphan, Janssen‐Cilag, Gilead and Amgen.
- Lucia Farina: advisory board for Janssen; lecturing for Abbvie.
- Marta Coscia: advisory board and lecturing from Janssen for Gilead, Abbvie, and esearch funding from Janssen and Karyopharm Therapeutics
- Robin Foà34: Editorial boards and/or speaker's bureau for Janssen, AbbVie, Amgen, Novartis, Roche, Pfizer.
- Paolo Ghia: honoraria from AbbVie, Adaptive, ArQule, BeiGene, Celgene/Juno, Dyname, Gilead, Janssen, Sunesis and research funding from AbbVie, Gilead, Janssen, Novartis.

## AUTHORS' CONTRIBUTIONS

AC, ARM, GMR, AP, MV, RF and PG designed the study and interpreted the data. AC, ARM, GMR, MG, LL, JNA, JMP, DMB, BTH, AW, NL, CST, RJ, FL, PMB, MS, APS, JJP, ARS, SJS, NNS, CSU, LR, EMO, AB, LT, MS, MM, AT, FI, GG, MD, LF, SM, FDR, MC, FRM, JdlS, AMP, IF, GC and PG collected data. AP and RC performed statistical analyses. AC and GMR wrote the manuscript. All the authors reviewed the manuscript for important intellectual contents and approved the final version of the manuscript.

## Supporting information

Fig S1

Table S1

Table S2

Table S3

## Data Availability

The data that support the findings of this study are available from the corresponding author upon reasonable request.
