# Supplementary material for: Efficacy of bendamustine and rituximab in unfit patients with previously untreated chronic lymphocytic leukemia. Indirect comparison with ibrutinib in a real‐world setting. A GIMEMA‐ERIC and US study
Source: Cancer Med. 2020 Sep 24;9(22):8468–79. doi: 10.1002/cam4.3470 (PMC7666748; doi:10.1002/cam4.3470)
Supplement: Supplementary file 1 — Fig S1 [file CAM4-9-8468-s001.doc]

Supplementary Figure 1 Time to Next Treatment (death was considered as a competitive risk)


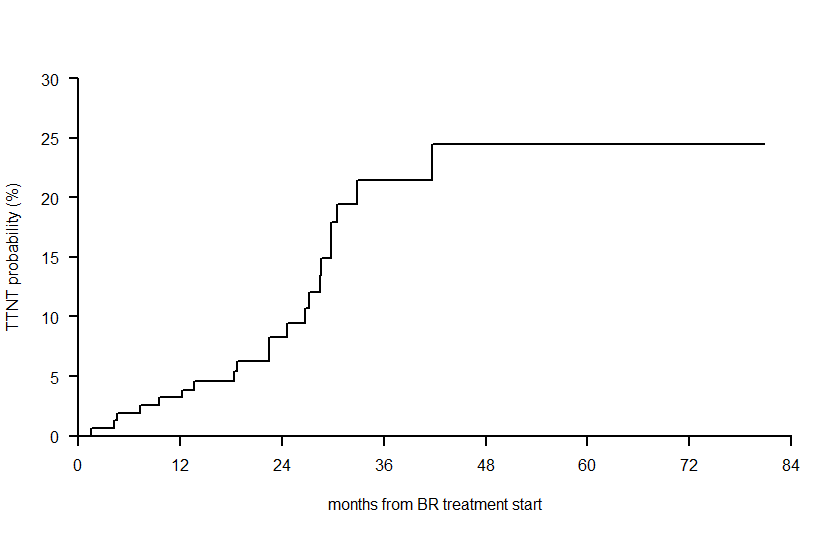


| **months** | **estimate** | **lower 95%CI** | **Upper 95%CI** |
| --- | --- | --- | --- |
| 12 | 3.2 | 1.2 | 6.9 |
| 24 | 8.3 | 4.3 | 13.9 |
| 36 | 21.5 | 13.1 | 31.1 |
