# Supplementary material for: Efficacy of bendamustine and rituximab in unfit patients with previously untreated chronic lymphocytic leukemia. Indirect comparison with ibrutinib in a real‐world setting. A GIMEMA‐ERIC and US study
Source: Cancer Med. 2020 Sep 24;9(22):8468–79. doi: 10.1002/cam4.3470 (PMC7666748; doi:10.1002/cam4.3470)
Supplement: Supplementary file 2 — Table S1 [file CAM4-9-8468-s002.doc]

Supplementary Table 1. ORR and univariate analyses

|  | **PD/SD/NR/Death n = 14 (%)** | **CR/PR n= 143 (%)** | **p** |
| --- | --- | --- | --- |
| age ≤65 / >65 years | 1 (7.1) / 13 (92.9) | 29 (20.3) / 114 (79.7) | 0.40 |
| stage †early/ intermediate-advanced | 3 (27.3) / 8 (72.7) | 50 (42.4) / 68 (57.6) | 0.51 |
| beta 2 microglobulin ≤3·5 / >3.5 mg/L | 0 (0.0) / 11 (100.0) | 19 (18.6) / 83 (81.4) | 0.25 |
| *IGHV* Mutated / Unmutated | 3 (60.0) / 2 (40.0) | 54 (49.5) / 55 (50.5) | 1.00 |
| 17p- and/or *TP53* mutated yes /no | 5 (50.0) / 5 (50.0) | 18 (14.9) / 103 (85.1) | 0.02 |

†early: Binet A

Legend: CR: complete response; PR Partial response; PD: progressive disease, SD stable disease, NR; no response.
