# Supplementary material for: Efficacy of bendamustine and rituximab in unfit patients with previously untreated chronic lymphocytic leukemia. Indirect comparison with ibrutinib in a real‐world setting. A GIMEMA‐ERIC and US study
Source: Cancer Med. 2020 Sep 24;9(22):8468–79. doi: 10.1002/cam4.3470 (PMC7666748; doi:10.1002/cam4.3470)
Supplement: Supplementary file 3 — Table S2 [file CAM4-9-8468-s003.doc]

Supplementary table 2. TTNT: univariate and multivariate analyses in the BR cohort.

|  | **Univariate** | | **Multivariate** | |
| --- | --- | --- | --- | --- |
|  | **HR (95% CI)** | **p** | **HR (95% CI)** | **p** |
| Age >65 vs ≤ 65 years | 2.47 (0.70-8.05) | 0.13 |  |  |
| Binet B-C vs Binet A | 1.99 (0.88-4.51) | 0.10 |  |  |
| beta 2 microglobulin ≥3.5 / <3.5 mg/L | 1.55 (0.54-4.50) | 0.41 |  |  |
| *IGHV* unmutated vs mutated | 1.99 (0.80-5.00) | 0.14 |  |  |
| 17p- and/or *TP53* mutated (no / yes) | 0.25 (0.10-0.63) | <0.01 | 0.30 (0.12-0.80) | 0.02 |
| CR-PR no vs yes | 10.65 (3.26-34.76) | <0.01 | 8.34 (3.59-19.37) | <0.01 |
