# Supplementary material for: Efficacy of bendamustine and rituximab in unfit patients with previously untreated chronic lymphocytic leukemia. Indirect comparison with ibrutinib in a real‐world setting. A GIMEMA‐ERIC and US study
Source: Cancer Med. 2020 Sep 24;9(22):8468–79. doi: 10.1002/cam4.3470 (PMC7666748; doi:10.1002/cam4.3470)
Supplement: Supplementary file 4 — Table S3 [file CAM4-9-8468-s004.docx]

Supplementary table 3. OS: univariate and multivariate analyses in the combined BR and ibrutinib cohorts.

|  | **Univariate** | | | **Multivariate** | |
| --- | --- | --- | --- | --- | --- |
| **Variable** | **HR (95% CI)** | **p** |  | **HR (95% CI)** | **p** |
| **Age (continuous variable)** | 1.10 (1.04 - 1.15) | <0.01 |  | 1.10 (1.04 - 1.15) | <0.01 |
| **Age >70 vs. ≤70 years** | 0.50 (0.20 - 1.24) | 0.14 |  |  |  |
| **Gender female vs male** | 0.73 (0.34 - 1.54) | 0.41 |  |  |  |
| **Time dx-trx* (continuous variable)** | 1.00 (0.99 - 1.01) | 0.92 |  |  |  |
| **interval dx-trx >36 vs <36 months** | 0.82 (0.40 - 1.68) | 0.59 |  |  |  |
| **IGHV (Mutated vs.  Not Mutated)** | 0.65 (0.20 - 2.10) | 0.47 |  |  |  |
| **Rai (continuous variable)** | 1.49 (1.01 - 2.18) | 0.04 |  |  |  |
| **RAI 3-4 vs 0-2** | 1.64 (0.71 - 3.76) | 0.24 |  |  |  |
| **11q- Yes vs. No** | 3.40 (1.10 - 10.52) | 0.03 |  |  |  |
| **Ibr vs. BR** | 0.50 (0.20 - 1.24) | 0.14 |  |  |  |

 *Time dx-trx: Interval between diagnosis and treatment
